# Discovering local patterns of co - evolution: computational aspects and biological examples

**DOI:** 10.1186/1471-2105-11-43

**Published:** 2010-01-22

**Authors:** Tamir Tuller, Yifat Felder, Martin Kupiec

**Affiliations:** 1 School of Computer Science, Tel Aviv University, Tel Aviv, Israel; 2 Department of Molecular Microbiology and Biotechnology, Tel Aviv University, Tel Aviv, Israel; 3 Sackler School of Medicine, Tel-Aviv University, Tel Aviv, Israel; 4 Faculty of Mathematics and Computer Science, Weizmann Institute of Science, Rehovot, Israel

## Abstract

**Background:**

Co-evolution is the process in which two (or more) sets of orthologs exhibit a similar or correlative pattern of evolution. Co-evolution is a powerful way to learn about the functional interdependencies between sets of genes and cellular functions and to predict physical interactions. More generally, it can be used for answering fundamental questions about the evolution of biological systems.

Orthologs that exhibit a strong signal of co-evolution in a certain part of the evolutionary tree may show a mild signal of co-evolution in other branches of the tree. The major reasons for this phenomenon are noise in the biological input, genes that gain or lose functions, and the fact that some measures of co-evolution relate to rare events such as positive selection. Previous publications in the field dealt with the problem of finding sets of genes that co-evolved along an entire underlying phylogenetic tree, without considering the fact that often co-evolution is local.

**Results:**

In this work, we describe a new set of biological problems that are related to finding patterns of *local *co-evolution. We discuss their computational complexity and design algorithms for solving them. These algorithms outperform other bi-clustering methods as they are designed specifically for solving the set of problems mentioned above.

We use our approach to trace the co-evolution of fungal, eukaryotic, and mammalian genes at high resolution across the different parts of the corresponding phylogenetic trees. Specifically, we discover regions in the fungi tree that are enriched with positive evolution. We show that metabolic genes exhibit a remarkable level of co-evolution and different patterns of co-evolution in various biological datasets.

In addition, we find that protein complexes that are related to gene expression exhibit non-homogenous levels of co-evolution across different parts of the *fungi *evolutionary line. In the case of mammalian evolution, signaling pathways that are related to *neurotransmission *exhibit a relatively higher level of co-evolution along the *primate *subtree.

**Conclusions:**

We show that finding local patterns of co-evolution is a computationally challenging task and we offer novel algorithms that allow us to solve this problem, thus opening a new approach for analyzing the evolution of biological systems.

## 1 Background

Co-evolution is the process by which two (or more) sets of orthologs exhibit a similar or a correlative pattern of evolution. Co-evolution can be measured in various ways; those most commonly used are: the similarity in absolute Evolutionary Rate (ER; dN; the rate of non-synonymous substitutions) or dN/dS (the rate of non-synonymous substitutions rate divided by the rate of synonymous substitutions) [1-3], correlative ER or dN/dS [4-6], and similarity in the pattern of protein presence in the proteomes of a set of organisms [7-9]. Detecting co-evolving sets of orthologs is an important matter since physically interacting proteins [4,5,10,11] and functionally related proteins [1,3,6,12,13] tend to co-evolve. Thus, an appropriate analysis of co-evolving genes can lead to a better understanding of the evolution of various cellular processes and gene modules (*e.g*. see [14] and [15]).

The most popular approach for detecting co-evolution is based on phylogenetic profiles [7-9]. It searches groups of orthologs with similar phyletic patterns. The main disadvantage of this approach is the fact that it totally ignores the topology of the organisms' evolutionary tree. A similar measure is the Propensity for Gene Loss (PGL) in evolution [12,13,16]. Genes with lower PGL have lower ER and tend to be essential for the viability of the organism. It has been proven recently [13] that orthologs with correlative PGL tend to be functionally related.

Another related measure for evolutionary distance is the difference between the average *dN/dS *or ER of pairs of orthologs [1-3]. Using this measure Marino *et al*. showed that there is a strong connection between the function of genes and their evolutionary rates [3]. All previous approaches for detecting co-evolution have not considered the fact that gene modules can exhibit strong patterns of co-evolution in some parts of the evolutionary tree while exhibiting a very weak signal of co-evolution in other periods of their evolution. There may be a number of reasons for this phenomenon.

First, evolving genes may gain or lose functions (see *e.g*. [17]); loss or gain of a new function can move an ortholog from one co-evolving module to a different one. Additionally, there may be differences in evolutionary pressure acting within ortholog groups in different parts of the evolutionary tree (see *e.g*. [18]). Second, the analyzed biological data may be noisy or partial in some portions of an evolutionary tree while it can have higher quality in other parts. In such cases, searching sets of orthologs with similar evolution along the *entire *phylogenetic tree may result in high false negative rates. Third, there are co-evolutionary problems that are local by definition. For example, genes tend to undergo positive selection in a small fraction of their history (see *e.g*. [19]). Thus, if we define co-evolution as a process in which a set of orthologs undergoes positive selection together, we should not expect that such type of co-evolution should span the entire phylogenetic tree.

**Figure 1:**
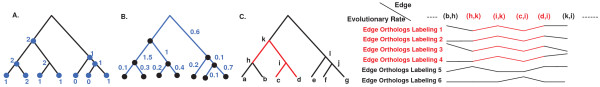
***A*. A hypothetic example of a node orthologous labeling which includes gene copy number in each node of the evolutionary tree**. *B*. A hypothetic example of an edge orthologous labeling which includes *dN/dS *along each edge of the evolutionary tree. *C*. The goal of the local co-evolutionary problem is to find large sets of orthologs that have similar patterns of evolution across large subtrees of the evolutionary tree.

The goal of this work is to study the *Local Co-Evolutionary *problem. Namely, given a phylogenetic tree and a set of vectors describing the evolution of orthologous sets along the evolutionary tree we aim to find sub-sets of orthologs with similar evolution along subtrees of the evolutionary tree (see Figure 1C). We formalize a new set of *Local Co-Evolutionary *problems, study their computational hardness and describe algorithms and heuristics for solving them. A simulation study shows that these algorithms give much better performances than popular bi-clustering algorithms for gene expression. Finally, we generate five relevant biological datasets and use our computational tools to analyze them. Three datasets include *dN/dS *and gene Copy Number (CN) of thousands of orthologs across evolutionary trees. The two other datasets include the *dN/dS *and CN related to hundreds of signaling pathways and protein complexes across evolutionary trees with dozens of nodes.

## 2 Definitions and Preliminaries

As was mentioned in the Introduction, in this work the aim is to find sets of orthologs with similar evolutionary along parts (subtrees) of the evolutionary tree. In this section, we formally define this problem. Furthermore, we define several measures of co-evolution and a few possible inputs to our problem.

Let *T *= (*V, E*) be a tree, where *V *and *E *are the tree *nodes *and tree *edges *respectively. In this work, we consider rooted binary phylogenetic trees (*i.e*. the degree of each node in the tree is either 1, 2, or 3), and all the trees that are described in this work are species trees. A node of degree 1 is named a *leaf*, a node with degree 3 is named an *internal node*, and the root has degree 2. A tree *T' *is a subtree of *T *if it is a connected subgraph in *T*. We denote such a relation by *T' *⊆ *T*. Note that by the above definition an internal node of a tree *T *can be a leaf in the subtree *T' *⊆ *T*.

A *Node Orthologous Labeling *(*NOL*) of a tree *T*, is a set of labels (real numbers) for each of the nodes in *T*; an *Edge Orthologous Labeling *(*EOL*) for a tree *T*, is a set of labels for each of the edges in *T *(see Figure 1). An Orthologous Labeling (*OL*, *i.e*. a *NOL *or *EOL*) of a tree reflects the evolutionary patterns along the tree. Thus, we also name the *OL *of a tree: *the evolutionary pattern along the tree*.

Let *S *denote a set of *OL*s in *T*, and let *S' *be a subset of *S*. Let *D*_*c*_(*S'*, *T'*) denote a measure for co-evolution along a subtree, *T' *⊆ *T*. Such a measure returns a real positive number which reflects how similar is the co-evolution of the *OL*s from *S' *along the subtree *T' *(0 reflects an identical evolution). Formally, we deal with versions of the following problem:

**Problem 1 ***Local Co-Evolution*

*Input: A phylogenetic tree, T *= (*V*, *E*), *a set of *NOL*s or *EOL*s, S *= [*S*_1_,.., *S*_*m*_], *two natural numbers, n'*, *m'*, *a real number, d, and a measure of co-evolution, D*_*c*_(.,.). *Question: Is there a subtree T' *= (*V'*, *E'*) ⊆ *T with *|*E'*| = *n'*, *and a subset S' *⊆ *S with *|*S'*| = *m'*, *such that D*_*c*_(*S'*, *T'*) ≤ *d *?

In the rest of this section we describe a few examples of *NOL*s and *EOL*s, and give a few examples of measures of co-evolution.

In this work, we analyzed one *NOL*:

(1) **Gene copy number of orthologs**, which is the number of paralogs of a given gene (from a certain orthologous group) in each node (genome and ancestral genome) of the evolutionary tree. In general, we can deal both with absolute values and discrete values of gene copy numbers. In the discrete case, we are only interested in whether a certain ortholog appears or not in each node of the evolutionary tree and not in the number of times it appears, while in the absolute value we do consider the number of times each ortholog appears in each node of the evolutionary tree.

We also analyzed two *EOL*s:

(1) **Non-synonymous substitution rate**, *dN*, **divided by the synonymous substitution rate, ***dS ***(*i.e*. ***dN/dS***)**. We examined absolute, discrete, and relative values of *dN/dS*. The absolute case is *dN/dS *(a positive real number) without additional processing. In the discrete case, we only consider three possibilities: dN/dS > 1 (positive selection, *dN/dS *> 1), dN/dS ≈ 1 (neutral selection, *dN/dS *≈ 1), or dN/dS < 1 (purifying selection, *dN/dS <*1). In the relative case, we perform an additional normalization of the dN/dS of each orthologous group by comparing them to the dN/dS of other orthologous groups. This is done by computing for each edge of the tree the rank of the dN/dS of an orthologous group among the dN/dS of all orthologous group.

(2) **Change in orthologous gene Copy Numbers (CN) **along the tree edges. In this case, we can check the exact changes or only the direction of the changes (*i.e*. if the copy number increases, decreases, or does not change along an edge).

**Figure 2:**
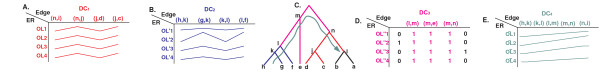
**Illustration of the four measures of local co-evolution (*A*. *D*_*c*1_, *B*. *D*_*c*2_, *D*. *D*_*c*3 _and *E*. *D*_*c*4_) along a hypothetical evolutionary tree (*C*.)**. *A*. In the case of *D*_*c*1_, the co-evolution score is high when values of all the *OL*s along a sub-tree are similar. *B*. In the case of *D*_*c*2_, the co-evolution score is high when values of all the *OL*s along a sub-tree are correlative (*i.e*. the *OL*s tend increase/decrease on the same branches). *D*. *D*_*c*3 _is used for finding a large subtree and a set of orthologs with identical labeling along most of a subtree. *E*. *D*_*c*4 _is used for finding sets of orthologs that have similar monotonic/non-monotonic decreasing/increasing evolutionary pattern along a path.

We analyzed the following measures of co-evolution (Figure 2; we usually give examples that are related to dN/dS but with the appropriate changes all the measures can be implemented on *NOL*s and on labelings that are related to CNs):

(1)  is the maximal *L*_1 _norm between all the pairs of  along the evolutionary subtree *T'. D*_*c*1 _measures the similarity of the absolute values in the *OL*s (see Figure 2A). Thus, orthologs that have similar *dN/dS*s along each branch of *T' *will have a significantly low *D*_*c*1_.

(2) , where *r *denotes the minimal Spearman correlation among all pairs of the *OL *of  along the edges or nodes of *T'*. Orthologs can differ in their average *dN/dS *but exhibit similar fluctuations in their ER (see Figure 2B). *D*_*c*1 _can not discover such pattern of co-evolution but *D*_*c*2_, as it finds sets of orthologs with correlative pattern of evolution, is suitable for this task.

(3)  where ℓ is a certain labeling. This measure is used for finding a large subtree and a set of orthologs with identical labeling along most of this subtree (see Figure 2C).

In this work, we used this measure for finding a subtree where a set of orthologs undergoes positive selection (*i.e. dN/dS >*1) together. To this end, we first performed a two-level discretization of the *dN/dS *values; one discrete level was assigned to the *dN/dS *above 1 and the second discrete level was assigned to the *dN/dS *below 1.

(4) **D**_**c4**_(**S'**, **T'**): In the case of *D*_*c*4_(*S'*, *T'*), we want to find a path along the evolutionary tree (*i.e. T' *is a path), and a set of OLs, *S'*, that have similar monotonic/non-monotonic decreasing/increasing evolutionary pattern along the path (see Figure 2D).

*D*_*c*4_(*S'*, *T'*) = *d *denotes that the maximal number of components of an orthologous labeling, *S*_*i *_∈ *S'*, that should be changed in order to fit them to the pattern that the path induces is less than *d*. This measure can be useful for discovering modules of orthologs that exhibit together acceleration or deceleration in their ER or *dN/dS *along a certain evolutionary path due to speciation.

## 3 Hardness Issues

Hardness issues that are related to the *Local Co-Evolutionary *problem appear in additional files 1 and 2. Specifically, in this note, we show that some versions of the problem are NP-hard, but in practice it seems that the *Local Co-Evolutionary *problem has a shorter running time than the *bi-clustering *problem which is highly used in the context of gene expression analysis. Furthermore, we show that there are versions of the *Local Co-Evolutionary *problem that can be solved by a fixed-parameter tractable (FPT) algorithm or by a polynomial algorithm.

## 4 Methods

### 4.1 Heuristics and Algorithms

This section includes a description of the two algorithms that we developed for finding local patterns of co-evolution. The goal of these algorithms is to find co-evolving sets with at least *m' OL*, that exhibit co-evolution score <*d *along a tree with more than *n' *edges. The threshold *d *determines the sizes of the subtrees in the solutions. It is easy to see that on average *OL*s corresponding to larger subtrees have higher co-evolutionary score. Thus, larger *d *will result in solutions with larger subtrees while smaller *d *will result in solutions with smaller subtrees.

**Figure 3:**
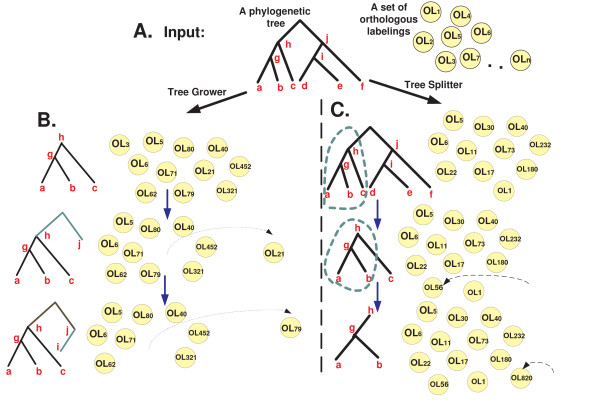
**An illustration of the two algorithms (see the text for more details)**. *A*. The input. *B*. The *Tree Grower *algorithm. *C*. The *Tree Splitter *algorithm.

We designed two main algorithms. The first algorithm, the *Tree Grower*, starts with sets of orthologs with similar patterns of evolution along small subtrees, and expands these initial trees while possibly decreasing the set of orthologs (Figure 3B). The second algorithm, the *Tree Splitter*, finds first sets of orthologs with similar pattern of evolution along the entire input tree, and recursively cuts edges from the initial tree while possibly increasing the sets of orthologs (Figure 3C).

As we demonstrate in the next sections, each of these algorithms has it own advantages. As the *Tree Grower *is a bottom up algorithm, it outperforms the *Tree Splitter *in finding sets of *OL*s that co-evolve along relatively small parts of the evolutionary tree. On the other hand, the *Tree Splitter *is better at finding sets of *OL*s that co-evolve along larger parts of the evolutionary tree.

#### 4.1.1 The Tree Grower Algorithm

Let *d*_*g *_<*d*, *m*_*g *_>*m'*, and *n*_*g *_<*n' *denote pre-defined parameters.

The first stage of the *Tree Grower *algorithm includes generating a collection of sets of *OL*s (seeds) that have a high co-evolutionary score along a small subtree (*e.g*. a subtree with around log(*n*) nodes or edges). The set of seeds was generated by the FPT procedure that we described in the previous sections, or by implementing K-means [20] on the *OL*s that are induced along each of the small subtrees. Formally, each set includes at least *m*_*g *_>*m' OL*s that have significant co-evolving score (*d*_*g *_<*d*) on small subtrees (trees that have less than *n*_*g*_edges).

**Figure 4:**
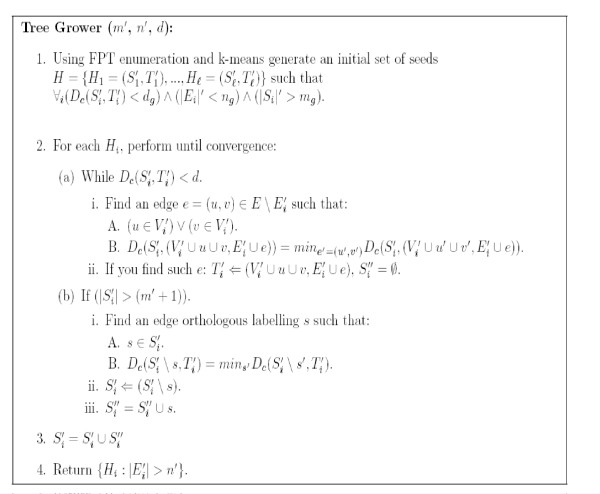
**The *Tree Grower *algorithm for the *Local Co-Evolution *problem with edge orthologous labelings**. A similar heuristic was used for the *Local Co-Evolution *problem with node orthologous labelings.

Next, the *Tree Grower *procedure greedily 'grows' solutions with larger subtrees that may have less *OL*s than in the initial seeds. This is done by increasing the size of the trees in the initial seeds while possibly decreasing the number of orthologs in each set. Each solution includes at least *m' *orthologs that have co-evolution scores better than *d *across a subtree with at least *n' *edges (see Figure 4 for exact details).

Let *f*_*c*_(|*E*|, |*S*|) denote the running time for computing *D*_*c*_(*S*, *T*). In the most general case, the running time of the *Tree Grower *on an input tree *T *= (*V, E*), a set of *OL*s, *S*, and initial set of seeds of size |*H*| is *O*((|*E*| + |*S*|)·|*S*|·|*E*|·|*H*|·*f*_*c*_(|*E*|, |*S*|)).

#### 4.1.2 The Tree Splitter Algorithm

Let *d*_*s *_>*d *and *m*_*s *_<*m' *denote pre-defined parameters.

In this case, by the FPT procedure and by K-means we first generated a set of clusters of *OL*s along the entire input phylogenetic tree. Each of the initial set of seeds includes all the edges of the tree but has relatively low number of orthologs (*m*_*s *_<*m'*), and high co-evolution score (*d*_*s *_>*d*).

**Figure 5:**
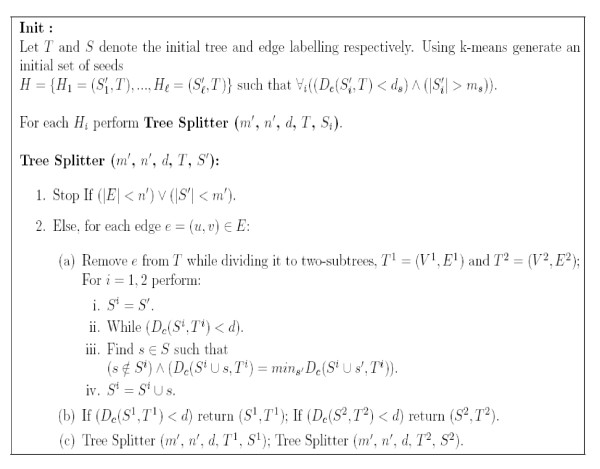
**The *Tree Splitter *algorithm for the *Local Co-Evolution *problem with edge orthologous labelings**. A similar heuristic was used for the *Local Co-Evolution *problem with node orthologous labelings.

Next, at each stage, the *Tree Splitter *algorithm cuts edges from the subtree related to each cluster while greedily increasing the size of the set of *OL*s that is related to the cluster. The outputs of the algorithm are co-evolving sets of orthologs (of size at least *m' *orthologs) that have co-evolution scores better than *d *across a subtree of size at least *n' *(see Figure 5). Let *K *denote the initial number of clusters; the running time of *Tree Splitter *is |*K*|·|*S*|·|*E*|·*f_c_*(|*E*|, |*S*|). The *Tree Splitter *algorithm is usually faster than the *Tree Grower*.

#### 4.1.3 The parameters used for the algorithms

In the case of the tree grower algorithm, the initial seeds were generated by performing k-means with *k *between 10 and the number of OLs divided by 10 (we filtered similar clusters), and by checking (extending) all possible paths in the tree. In the case of the tree splitter algorithm, the initial seeds included all the branches of the tree (and the OLs as before). In the case of the tree grower, *d *was at most *x *= %30 higher than *d*_*g*_, in the case of the tree splitter, *d *was at least *x *= %30 lower than *d*_*s*_. The minimal size of each solution appears in the corresponding supplementary table (see additional files 3, 4, 5, 6, 7, 8, 9 and 10 with the results).

### 4.2 The Random Trees Used in the Simulation

The random trees used in the simulation were generated by the following algorithm:

Generate a random tree:

• Start with the set of nodes corresponding to the tree's leaves, *L*.

• While *|L| >*1:

• - Choose two random nodes, *l*_1 _and *l*_2_, from *L*.

• - Merge these leaves to a new node, *l*_1,2 _(corresponding to an internal node of the tree).

• - *L *← *L*/{*l*1 ∪ *l*2} ∪ *l*_1,2_

It is easy to see that each such a step can describe an internal node (*l*_1,2_) whose two children are the two nodes (leaves or internal nodes) that were merged to generate it (*l*_1 _and *l*_2_).

### 4.3 P-values and GO Enrichments

#### 4.3.1 P-values

Empirical p-values for a co-evolving set of *m' OL*s over subtrees of size *n'*, when the input includes *m OL*s along a tree of size *n*, was computed by the following permutation test: 1) Generate *N *random permutated versions of the input, each permutated version is the result of *O*(*n · m*) single random permutations of the *OL*s of the original input. 2) Implement the algorithms for finding co-evolving sets on these random inputs. 3) Compute the fraction of times the algorithms found a co-evolving set with larger properties (*m' *and *n'*) than the original one. In this work we used *N *= 100 to filter solutions when we analyzed the biological datasets.

#### 4.3.2 GO-enrichment

GO enrichment of the co-evolving sets was computed using the GO ontology of *S. cerevisiae *(downloaded from the *Saccharomyces *genome database, http://www.yeastgenome.org/) and *H. Sapiens *(downloaded from EBI - BioMart, http://www.biomart.org/). We used the algorithm of Grossmann *et al*. [21] for detecting over-represented GO terms. All the *S. cerevisiae *or the *H. Sapiens *genes respectively were used as reference for the enrichments calculations. We decided to use a global background (the entire gene set of *H. sapiens *and *S. cerevisiae*) for the enrichment computation since we believe that part of the signal of co-evolution can appear in the analyzed datasets themselves. For example, *OL*s that exhibit change(s) in their copy number (see, for example, section 4.5.2) may have higher chance to co-evolve. Thus, the enrichments reported in this paper should be related both to the methods that we used and the datasets we analyzed.

### 4.4 Implementation

The software for the algorithms (*Tree Grower *and *Tree Splitter*) was written in C++, and the implementation run on regular PCs (Pentium M, 1400 MHz with 512 MB of RAM, and with Windows XP) and is available upon request.

### 4.5 Biological inputs

**Figure 6:**
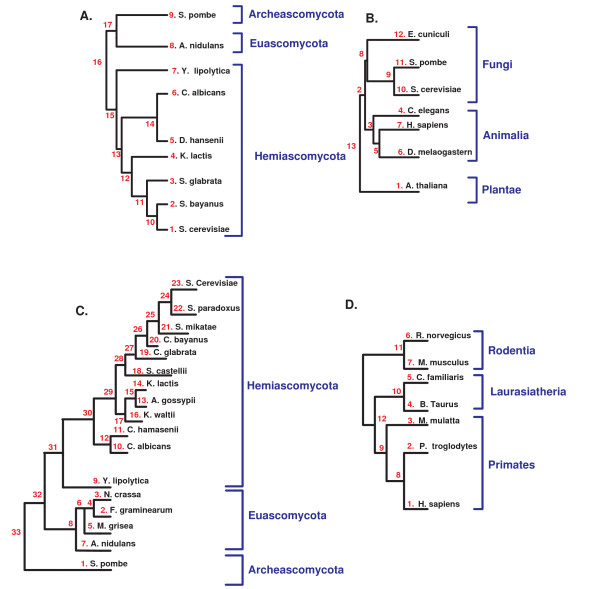
**The phylogenetic trees of the analyzed biological datasets**: *A*. The small fungi dataset, *B*. The eukaryote dataset, *C*. The large fungi dataset, *D*. The mammalian dataset.

We analyzed five biological datasets: 1) relative *dN/dS *of 1, 372 orthologous sets (12, 348 genes) along the phylogenetic tree of nine fungi (Figure 6A); we named this dataset the small *fungi dN/dS *dataset. 2) Gene copy number of 6, 227 orthologous sets (56, 043 genes) along the same phylogenetic tree of the nine fungi (Figure 6A); we named this dataset the *fungi CN *dataset. 3) gene copy number of 4, 851 orthologous sets (33, 957 genes) along the phylogenetic tree of seven eukaryotes (Figure 6B); we named this dataset the *eukaryote *dataset. 4) The mean changes in the copy number of 190 complexes along the phylogenetic tree of 17 fungi (Figure 6C); we named this dataset the large *fungi *complexes dataset. 5) The mean *dN/dS *of 85 signaling pathways along the phylogenetic tree of seven mammals (Figure 6D); we named this dataset the *mammalian *signaling pathway dataset.

The analyzed organisms included eukaryotes and in particular fungi; horizontal gene transfer events are very rare in these organisms. Thus, the methods used for inferring the ancestral families of orthologs, which assume only gene deletions and duplications, should reliable.

**Figure 7:**
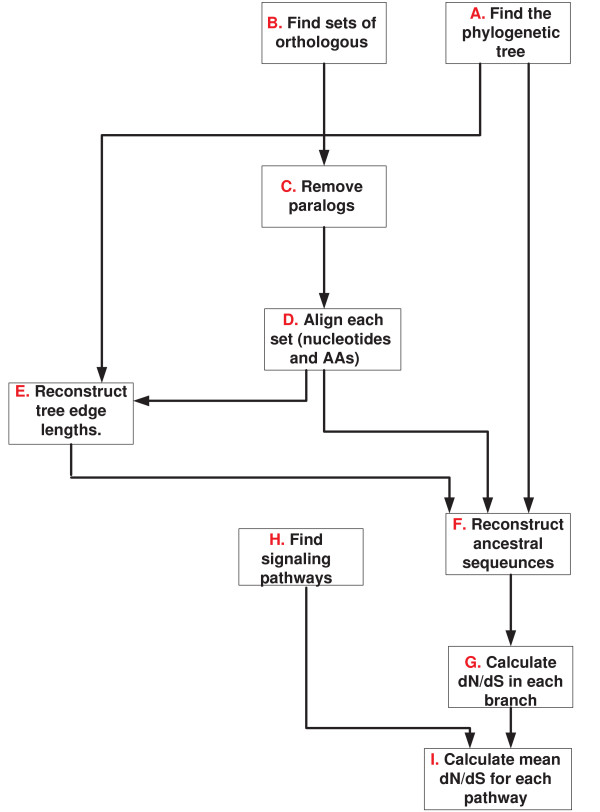
**The different steps in generating the small fungi dN/dS dataset and the mammalian signaling pathway dataset**.

**Figure 8:**
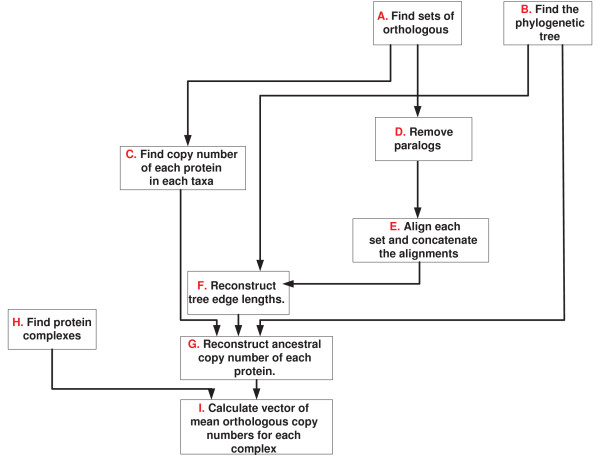
**The different steps in generating the small fungi CN dataset, the eukaryote CN dataset, and the large fungi complexes dataset**.

The following subsections include additional details about each of these inputs. Figures 7 and 8 describe the protocol used to generate the biological inputs.

#### 4.5.1 The small *fungi *dN/dS dataset

The small *fungi dN/dS *dataset was downloaded from [6]. The major stages in generating this dataset included identifying the phylogenetic tree, generating sets of orthologs without paralogs, aligning these sets, using maximum likelihood for reconstructing the ancestral genes of these orthologs (the sequences at the internal nodes of the phylogenetic tree), and using these orthologs and ancestral genes for computing ranked *dN/dS *values along each branch of the phylogentic tree (as we described in section 2; see also see steps *A *- *G *in figure 7).

#### 4.5.2 The small *fungi CN *dataset

The small *fungi CN *dataset was downloaded from [6]. This dataset includes sets of orthologs that exhibit at least one change in their corresponding gene copy number along the phylogenetic tree. The ancestral copy numbers for each of these sets were reconstructed by maximum likelihood. The gene copy number and ancestral copy number induce a set of *NOL*s that can further be translated to a set of *EOL*s (as we have described in section 2; see steps *A *- *G *in figure 8).

#### 4.5.3 The *eukaryote CN *dataset

This dataset includes orthologs from seven Eukaryotes whose phylogenetic tree appear in figure 6B. The set of orthologs were downloaded from the COG database [22]http://www.ncbi.nlm.nih.gov/COG/. The ancestral copy numbers were reconstructed by CAFE' [23]. To this end, we used the edge lengths estimations and phylogeny from the work of Hedges *et al*. [24]. Finally, using the copy number in each internal node, we computed the change in copy number for orthologous set along each edge to get a set of *EOL*s (see steps *A *- *G *in figure 8).

#### 4.5.4 The large *fungi *complexes dataset

This dataset includes the mean CN of complexes in 17 fungi whose phylogenetic tree appears in Figure 6C

The vectors of copy number and ancestral copy number of orthologs at each node of the large *fungi *phylogenetic tree (Figure 6C) were downloaded from [14]. The complexes of *S. cerevisiae *were downloaded from the *Saccharomyces *genome database http://www.yeastgenome.org/ and appear in additional file 3.

For each complex, we computed the mean copy number of its genes in each internal node and each leaf of the large fungi tree (Figure 6C). The input to our algorithm was a set of *EOL*s corresponding to the mean change in the complexes copy number along the edges of the evolutionary tree. Figure 8 describes this protocol used to generate the input.

#### 4.5.5 The *mammalian *signaling pathway dataset

Figure 7 describes the protocol used to generate this input. At the first stage, we computed the *dN/dS *of mammalian genes along each branch of their evolutionary tree (see Figure 6D). To this end, we downloaded the orthologous groups of the seven mammals that appear in figure 6D from EBI - BioMart Homology (BioMart November 2007). We considered only sets that include orthologs in all these species. Sets of homologs that did not include exactly one representative in each organism were removed from our dataset, to filter out paralogs and avoid potential errors in evolutionary rate estimation due to duplication events.

In the next step, stop codons were removed from each gene and the genes were translated to sequences of amino acids. The corresponding amino acid sequences of each orthologous gene set were aligned by CLUSTALW 1.83 [25] with default parameters. By using amino acids as templates for the nucleotide sequences and by ignoring gaps we generated gap-free multiple alignments of the three orthologous proteins in each orthologous set and their corresponding coding sequences.

Given the alignments of each set of orthologs and given the phylogenetic tree of the seven mammals (see Figure 6D), we used the codeml program in PAML for the joint reconstruction of ancestral codons [26] in the internal nodes of the phylogenetic tree. This reconstruction induced the sequence of the ancestral proteins and their corresponding ancestral DNA coding sequences. We hence obtained sets of 12 sequences; 7 from the previous step (corresponding to the 7 leaves of the phylogenetic tree) plus 5 reconstructed sequences of the internal node of the phylogenetic tree. We denote such a set of 12 sequences a complete ortholgous set. For each complete ortholgous set, we computed the dN (the rate of non-synonymous substitutions) and dS (the rate of synonymous substitutions) along each branch of the evolutionary tree by the *y*00 program in PAML [27,28].

In the second stage, we computed the mean *dN/dS *of the genes corresponding to each of 85 signaling pathways. The set of genes that appears in each pathway was downloaded from Ingenuity Pathways Analysis web-software http://www.ingenuity.com and is depicted in additional files 4.

## 5 Results and Discussion

### 5.1 Synthetic inputs

For evaluating the performances of our algorithms we designed the following simulation: 1) We generated random trees with 12 - 52 nodes by random hierarchical clustering of the trees' leaves, and generated random sets of 1000 - 3000 *OL*s that are related to these trees (see the Methods section). The labelings were sampled from the uniform distribution *U *[0, 3].

**Figure 9:**
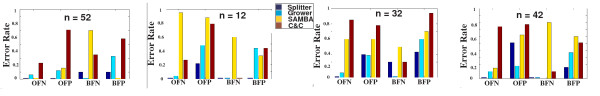
**Simulation study of the algorithms**. The figure depicts the average OFP, OFN, BFP, and BFN of the two algorithms (the *Tree Grower *and the *Tree Splitter*), and two bi-clustering algorithms (SAMBA and *C*&*C*) for different sizes of input trees (*n *is the number of nodes in the input trees). For each size of input trees we averaged the error rates of 100 simulations.

2) In these random inputs, we "planted" solutions, which are *OL*s (with 100 - 300 orthologs) that have high co-evolutionary score (identical co-evolution) in large subtree (*e.g*. 5 - 20 nodes) of the input tree. We added additive noise with uniform distribution *U*[-0.15, 0.15] to each component of the "planted" solutions.

3) We implemented the two algorithms, *Tree Grower *and *Tree Splitter*, on these inputs. 4) Currently there are no other algorithms that were designed specifically for discovering local patterns of co-evolution. Thus, we compared the performances of the algorithms to two popular bi-clustering algorithms (SAMBA [29] and the algorithm of Cheng and Church (*C*&*C*) [30]). To this end, we used two measures of performances: False Positive (FP) rate, which is the fraction of orthologs (OFP) or tree branches (BFP) in the output that are not part of a 'planted' solution, and False Negative (FN) rate, which is the fraction of orthologs (OFN) or tree branches (BFN) in the 'planted' solution that do not appear in the output. Figure 9 includes a summary of the simulation study. As can be seen, the performances of our algorithms are very good and far exceed the performances of the competing bi-clustering algorithms. For example, when considering *all *the synthetic inputs, the average OFN, OFP, BFN, and BFP of the *Tree Splitter *are 0.002, 0.25, 0.07, and 0.14 respectively. For comparison, the average OFN, OFP, BFN, and BFP of the algorithm of *C*&*C *are 0.52, 0.76, 0.16, and 0.61 respectively. This result justifies designing algorithms that are specific for solving the *co-evolutionary *problem, instead of using general bi-clustering algorithms.

Finally, our simulation showed that there are many inputs where the *Tree Splitter *algorithm outperforms the *Tree Grower *algorithm. However, there are cases where the *Tree Grower *gave better results (the intuition for this phenomenon was given in section 3.1). Thus, we employed both algorithms in the biological analysis.

### 5.2 Biological Inputs: Results and Discussion

In this section, we describe our main biological findings. The full lists of all the co-evolving sets that were found along with their local co-evolutionary patterns, and their functional enrichments appear in additional files 5, 6 and 7. The two main goals of this section are: 1) to describe a variety of biological examples that can be analyzed by our approach; 2) to depict some new biological insights related to this analysis.

The biological datasets describe the evolution of diverse sets of organisms and *OL*s, along different time ranges. The Eukaryote dataset includes both multicellular and unicellular organisms and describes evolution along 1642 million years. The fungi are unicellular organisms that appeared 837 million years ago. The mammals are multi-cellular organisms that appeared 197 million years ago (see [16,31] for the divergence times of the different phylogenetic groups).

In the case of the fungi, we analyzed both *dN/dS *and CN. The *dN/dS *dataset includes conserved *OL*s that have exactly one ortholog in each organism while the fungi CN dataset includes *OL *with varying number of orthologs in each organism (see section 4.5). In the case of the eukaryote dataset, we analyzed only CN. In Addition to the analysis of *OL*s we also analyzed the local co-evolution of mammalian signaling pathways (based on *dN/dS*) and fungi complexes (based on CN; see section 4.5).

The rest of this section includes comparisons between the different measures of co-evolution and a summary of our findings in each of the biological datasets. As mentioned in the methods section, the reported signals of co-evolution can be attributed both the different datasets that we analyzed and/or our computational approach. The fact that some of the signals appear in more than one of the analyzed dataset demonstrates that these signals are very robust. On the other hand, the fact that some enrichment appear in only part of the datasets may be attributed to the fact that the sets of *OL*s in each database are different, to the different measures of co-evolution that was used, and/or to the different type of *OL*s that was used.

#### 5.2.1 Comparison between the Different Measures of Co-evolution

**Figure 10:**
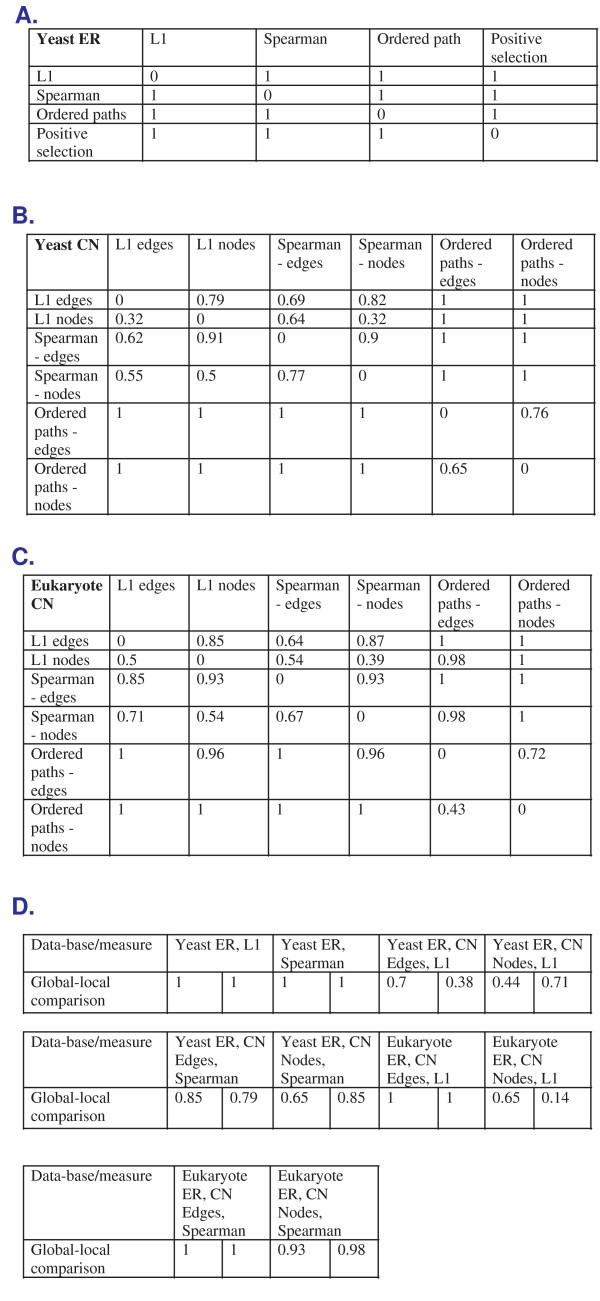
**Comparison between the different measures of co-evolution (*A*. - *C*.) and comparison between local and global co-evolution (*D*.)**. *A *- *C*: each table corresponds to one dataset, and each cell in these three tables corresponds to a comparison of two measures. These cells contain the fraction of the results that are not identical when comparing the corresponding plots of our approach; by our definition, 0 denotes identical sets of results while 1 denotes completely non-identical sets of results. *D*.: each table corresponds to one dataset, and each cell in these three tables corresponds to a global-local comparison of one measure.

The purpose of this subsection is to compare the different measures of co-evolution that are described in this work and to show that they are not redundant. To this end, we first compared the local co-evolutionary patterns found by the different measures of co-evolution. We defined two sets of co-evolving *OL*s to be identical if they have at least 70% similarity (measured by the corresponding Jaccard coefficient [32]) both when considering their *OL*s and when comparing the corresponding set of branches on which they co-evolve. Figures 10A, B, and 10C include such a comparison. Each table corresponds to one dataset, and each cell in these three tables corresponds to a comparison of two measures. These cells contain the fraction of the results that are not identical when comparing the corresponding plots of our approach. By our definition, 0 denotes identical sets of results while 1 denotes completely non-identical sets of results. As can be seen, the values in most of the cells are much closer to 1 than 0, demonstrating that the different measures of co-evolution are not redundant.

**Figure 11:**
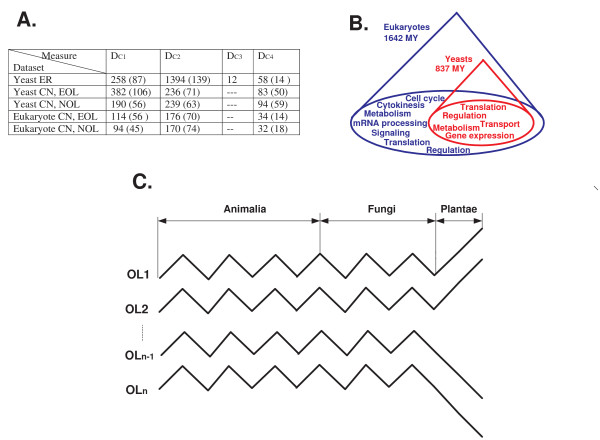
**A. Summary of the biological results**. The number of local co-evolving groups and the number of enriched co-evolving groups (in brackets) that were found in each of the biological datasets according to each of the co-evolution measures. B. A global view at the co-evolving functions (GO groups) in the yeast and the Eukaryote datasets, and the appearance time of each of the analyzed biological groups. C. An illustration of the break in co-evolution along the subtree of the *Plantae*.

Our approach can detect *regions *in the evolutionary tree where sets of orthologs exhibits co-evolution. By definition, this can not be done by clustering; we demonstrate this point in the next sections (see, for example, section 5.2.3). In this section, we demonstrate that also the *OL*s found by local and global approaches are different. To this end, we compared the results found by our approach to those obtained by a global clustering (*k*-means with various values of *k*). In this case, we only compared the *OL*s in each solution and used the same definition as described above. We compared the global and local results for each measure in each dataset. Figure 11D includes such a comparison. Again, as can be seen, the values in most of the cells are much closer to 1 than 0, demonstrating that many of the results found by our *local *approach can not be detected by a *global *clustering.

#### 5.2.2 Local Co-Evolution of Cellular Processes: A Global View

The small *fungi dN/dS *dataset, the small *fungi CN *dataset, and the *eukaryote CN *dataset relate to orthologs (single genes) and not to complexes/pathways as the other two datasets. Thus, it is possible to compute functional enrichment for the resulting sets that co-evolve locally (Methods, subsection 4.3.2).

A summary of these results appears in Figures 11A, B and 13. As can be seen, 10% - 56% of the co-evolving sets that we found are functionally enriched. This fact demonstrates that groups of genes with similar functionality tend to undergo local co-evolution.

Figure 11B depicts the main GO functions that were enriched in the co-evolving sets of *OL*s in each of the three datasets. As can be seen, our analysis shows that there are cellular processes, such as metabolism and regulation, that exhibit co-evolution in all the three datasets.

**Figure 12:**
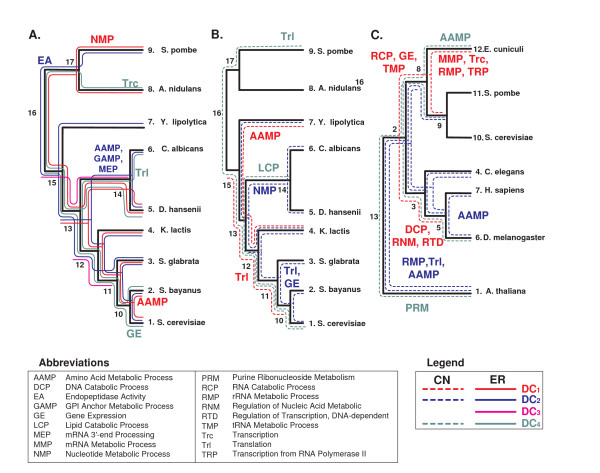
**A detailed description of the co-evolving sets of *OL*s, in the three biological datasets, enriched with metabolic and regulatory GO functions**: *A*. Yeast *dN/dS*, *B*. Yeast *CN*, *C*. Eukaryote *CN*. We marked regions in the trees where we detected co-evolving sets of *OL*s that are enriched with the aforementioned GO functions (see the abbreviation list in the middle of the figure). We used different colors (see the legend above) to distinguish between the different types of co-evolution. Dashed lines correspond to *CN *based co-evolution (*EOL *or *NOL*), and continues lines correspond to *dN/dS *based co-evolution (*EOL*).

Figure 12 includes a concentrated view on the co-evolution of the cellular processes that are related to metabolism and regulation in the three biological datasets. The figure depicts the regions in the evolutionary trees where we detected co-evolving sets of *OL*s that are enriched with metabolic and regulatory GO functions. This figure also includes information about the corresponding measures of co-evolution that were used for detecting each of the co-evolving sets of *OL*s. As can be seen, the fact that these two groups of cellular functions exhibit local pattern of co-evolution is robust to the type of the *OL*, the measure of co-evolution, and the input dataset used.

Additional file 6 includes a comparison between the co-evolving sets of *OL*s found by our approach and by SAMBA for the Fungi dN/dS dataset; it shows that many cellular functions were found to be enriched by our approach but not by SAMBA.

#### 5.2.3 Fungi Copy Number and dN/dS

**Figure 13:**
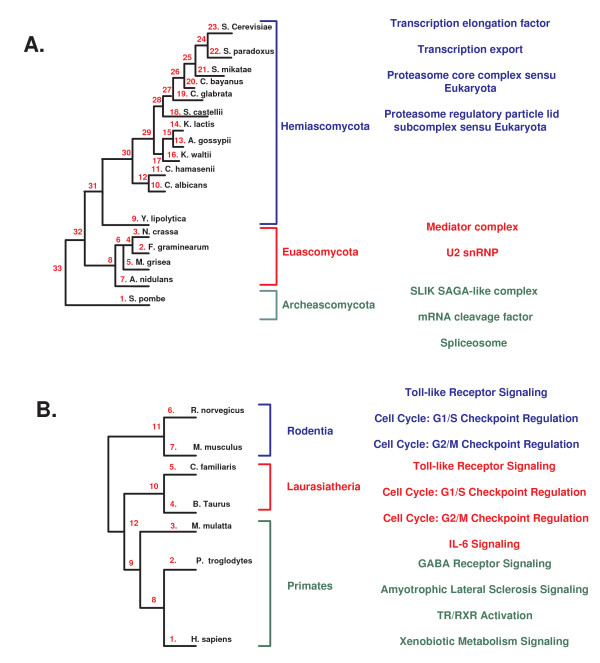
***A*. The complexes that exhibit relatively higher level of co-evolution in different parts of the Fungi tree**. *B*. The pathways that exhibit relatively higher level of co-evolution in different parts of the mammalian tree.

The two fungi datasets are interesting since they enable us to compare the two types of co-evolution: co-evolution via similar/correlative *dN/dS *(Figure 13A), and evolution via similar/correlative gene copy number (Figure 13B). Many metabolic cellular functions (*e.g*. metabolism of amino acids), and cellular functions that are related to regulation (*e.g*. translation) exhibit local co-evolutionary patterns both via changes in copy number and via changes in *dN/dS*. Though the GO enrichments that appear in Figure 13A and in Figure 13B are similar, it is important to note that the *OL*s (and thus the co-evolving sets of *OL*s) in the two cases are completely different. This fact emphasizes the centrality of these processes in the fungi evolution.

One explanation of this phenomenon is the fact that fungi datasets includes both anaerobic organisms (*S. cerevisiae*, *S. bayanus *and *S. glabrata*) and aerobic organisms (*A. nidulans*, *C. albicans*, *D. hansenii*, *K. lactis*, and *Y. lipolytica*) [33]; and the switch between these two types of metabolism required the co-evolution of various metabolic processes.

We discovered two regions where many of the fungal genes underwent positive selection. By definition, such regions in the evolutionary tree can not be discovered by global clustering methods. The larger set of *OL*s (554 orthologs) exhibits positive selection along the branch (11, 12) (see Figure 13A) probably following the whole genome duplication event that has occurred at this bifurcation [34]. This whole genome duplication event probably served as a driving force underlying this burst of positive selection, by relaxing the functional constraints acting on each of the gene copies (see for example [35]). Interestingly, this branch also partites the fungi into two groups, anaerobic and aerobic, that were mentioned above. This fact further supports the centrality of metabolism in fungi evolution.

Another set of *OL*s (11 orthologs) exhibits positive selection along the subtree with the nodes 13, 14, and 15 (see Figure 13A). The branch between nodes 13 and 14, leads to a subgroup (*D. hansenii *and *C. albicans*) that evolved a modified version of the genetic code [36], and the branch between nodes 13 and 15 leads to *Y. lipolytica *(which is a sole member in one of the three taxonomical clusters of the *Saccharomycotina *[37]). All the results for these datasets appear in additional file 5 and additional file 7.

#### 5.2.4 Eukaryote Copy Number

As mentioned, this biological dataset gives a wider evolutionary view than the fungi datasets. Cellular processes that are related to metabolism, signaling, and mRNA processing exhibit co-evolutionary patterns along this dataset (see Figures 11B and 13). One striking phenomenon is that many of these co-evolving sets (87%) exhibited co-evolution (according to all the measures of co-evolution) along the subtrees of the *Animalia *and *Fungi*, and excluding the subtree of the *Plantae *(see illustration in Figure 11C).

It is possible that this result is partially related to the fact that the analyzed subtrees of the *Plantae *included only one organism with relatively high evolutionary distance from other organisms. However, we also found two possible biological explanations for this phenomenon: First, many gene modules changed their functionality after the split between the *Plantae *and the two other groups (*Animalia *and *Fungi*). Cases where homologous protein complexes in *Plantae *and *Animalia *have rather different functions were reported in the past. For example, the *COP9 signalosome*, a repressor of photomorphogenesis in *Plantae*, regulates completely different developmental processes in *Animalia *[38,39]. Our analysis, however, may suggest that this is a wide scale phenomenon.

Second, it is possible that there is a relatively higher rate of changes in the protein-protein interactions along the split between the *Plantae *and the two other groups (*i.e*. more pairs of protein gain/lose new interactions). Thus, these results suggest that the protein-protein interaction network of *Plantae *may be relatively different from that of the other groups (see [40] for a comparison of protein-protein interaction networks). To the best of our knowledge, an alignment of the protein interaction network of a plant and organisms from the other two groups has not been performed yet. When such an alignment will be performed, it will be possible to check this hypothesis.

All the results for these datasets appear in additional files 8 and additional files 7.

#### 5.2.5 Co-Evolution of Cellular Functions

The functional enrichments of the co-evolving *OL*s can teach us about functional interdependencies between cellular functions and about the co-evolution of cellular functions. We found many subtrees where sets of *OL*s that are enriched with various GO functions exhibited co-evolution. For example, *Translation *and *Gene expression *exhibited a copy number based co-evolution in the fungi subtree that is under internal node 12 (Figure 12B), as expected from two coordinated biological processes in charge of producing RNA or proteins from the corresponding genes (DNA sequences).

Additional cellular processes showed coordinated evolution. For example, *Translation *and *Amino acid metabolic process *exhibited co-evolution in the Eukaryotes (Figure 12C) in the subtree that included nodes 1, 2, 3, 4, 5, and 8 (as detected by copy number variations). The link between these two processes is probably not direct. A possible explanation is that the evolution of the metabolism of various Amino Acids (*AA*) altered the composition of the *AA *pool in the fungi cell. These changes were then followed by a corresponding evolution of the translation machinery to cope with the new *AA *pool.

#### 5.2.6 Co-Evolution of Fungal complexes

We implemented our approach to find groups of complexes that exhibit correlative (Spearman Correlation) patterns of co-evolution along parts of the Fungi evolutionary tree (Figure 13A; see the Method section).

To discover complexes that co-evolve with other complexes in specific parts of the phylogenetic tree, we divided the evolutionary tree into the three parts that are marked in Figure 6C (*Hemiascomycota*, *Euascomycota*, and *Archeascomycota*). Then, we computed for each complex the number of solutions (co-evolving groups) that include it in each of these three parts of the tree (all the results appear in additional files 9). We focused on complexes whose co-evolution with other complexes is time dependent (*i.e*. it is relatively higher in a narrow part of the evolutionary tree).

We found that several complexes exhibit different levels of co-evolution with other complexes along different parts of the evolutionary tree. For example, the complexes: *Transcription elongation factor *and *Transcription export *which are important for mRNA production, as well as the *Proteasome core complex sensu Eukaryota *and *Proteasome regulatory particle lid subcomplex sensu Eukaryota*, in charge of protein degradation, exhibit relatively higher level of co-evolution in the subtree of the *Hemiascomycota*. These complexes affect general protein amounts in the cell at two different levels, transcription (mRNA formation) and protein stability (protein degradation). In the sub-tree of the *Euascomycota *we see co-evolution of the *Mediator complex *and the *U2 snRNP*. These two complexes affect mRNA level by influencing the rate of transcription and the rate of splicing, respectively. Finally, the *SLIK SAGA-like complex*, encoding a chromatin remodelling complex, as well as the *mRNA cleavage factor *and the *Spliceosome*, involved in mRNA processing, exhibit relatively higher level of co-evolution in the subtree of the *Archeascomycota*.

Notably, all the complexes whose co-evolution was enriched in specific branches of the tree are involved in basic gene expression processes at all possible levels (mRNA creation, stability and processing, protein creation and stability). A recent work of Man and Pilpel [33] showed that differential translation efficiency of orthologous genes can produce phenotypic divergence of Fungi. Our results may suggest a similar and wider picture where the co-evolution of various gene expression processes is involved in phenotypic divergence.

#### 5.2.7 Co-Evolution of Mammalian Signaling Pathways

Similarly to the previous subsection, we implemented our approach to find groups of signaling pathways that exhibit correlative (Spearman Correlation) and absolutely similar (*L*_1 _norm) pattern of co-evolution along parts of the mammalian evolutionary tree (Figure 13B; see the Methods section).

To discover co-evolution of specific pathways in specific parts of the phylogenetic tree, we divided the evolutionary tree into the three parts that are marked in Figure 6D (*Rodentia*, *Laurasitheria*, and *Primates*). Then, we computed for each pathway the number of solutions (co-evolving groups) that include that pathway in each of these three parts of the tree (all the results appear in additional file 10). We focused on those signaling pathways whose co-evolution is time dependent (*i.e*. it is relatively higher in a narrow part of the evolutionary tree).

In this case, we found that in general pathways exhibit relatively homogenous levels of co-evolution along different parts of the evolutionary tree. However, also in this case, for the *L*_1 _norm, some of the pathways exhibit accelerated levels of co-evolution in particular branches. For example, the pathways *Toll-like Receptor Signaling*, a pathogen-associated pattern recognition receptor, *Cell Cycle: G1/S Checkpoint Regulation*, and *Cell Cycle: G2/M Checkpoint Regulation *exhibit relatively higher levels of co-evolution in the subtrees *Rodentia *and *Laurasitheria*. Interestingly, in the latter subtree co-evolution can also be seen between these pathways and *IL-6 Signaling*, which plays a central role in inflammation. The association between basic cellular checkpoints and the response to external insults such as pathogens is intriguing and deserves further investigation.

Finally, in the subtree of the *Primates *we observe co-evolution of pathways related to neurotransmission and neuronal evolution (*e.g. GABA Receptor Signaling*, the main inhibitory neurotransmitter in mammalian CNS, *TR/RXR Activation*, related to activation of the thyroid hormone, and *Amyotrophic Lateral Sclerosis Signaling*, a disorder of the motor neurons).

## 6 Conclusions

In this work we carried out a large-scale analysis of local co-evolution. As some of these problems are NP-hard, we suggested two heuristics for solving them. We showed that the different measures of co-evolution are non-redundant. Finally, we demonstrated the biological significance of the local co-evolutionary problems through the analysis of five biological datasets. The goal of this part was to demonstrate how our computational tools can be used in practice.

In the future, we intend to extend this work in four directions. First, in this work, we showed that the local co-evolution is NP-hard when using *D*_*c*3 _as measure of co-evolution. It is important to show that detecting local co-evolution according to the other measures of co-evolution is also NP-hard. Second, in this work we described two heuristics for solving co-evolutionary problems. These heuristics gave very encouraging results in the simulation study. However, as we believe that better algorithms are within reach, we plan to spend more time in designing faster and more accurate algorithms for solving these problems. A related open problem is to find approximation algorithms for solving at least some of the co-evolutionary problems mentioned.

Third, in this work, we decided to demonstrate our approach by focusing on four typical versions of the *Local Co-Evolutionary *problem. However, the concept that was described here can be used for solving both more specific queries (*e.g*. finding co-evolving sets of *OL*s along a subtree that includes at least one leaf) and more general ones (*e.g*. a *joint *analysis of *dN/dS *and copy number of orthologs across a phylogenetic tree).

Finally, generating biological inputs for local co-evolutionary problems is a non-trivial task (see section 4.5 and [6,14,22]) as it includes dozens of preprocessing steps that should be performed properly. We plan to use our approach for studying co-evolution across the entire tree of life. To this end, we intend to generate the phylogenetic tree and the *OL*s of hundreds of organisms (Archaea, Bacteria, and Eukaryota), and to analyze this input by our approach.

## Authors' contributions

YF and TT participated in the design and execution of the study; TT and MK analyzed the results; TT and MK participated in the preparation of this manuscript. All authors read and approved the final manuscript.

## Supplementary Material

Additional file 1 **Supplementary Note**. 1 Hardness Issues. Click here for file

Additional file 2 **Supplementary Figure 1**. The *Bi-clustering *problem is identical to the *Local Co-Evolution *problem when the degree of the phylogenetic tree is unbounded. Click here for file

Additional file 3 **Supplementary Table 1**. List of Fungi complexes and the result patterns of co-evolution for the fungal complexes. Click here for file

Additional file 4 **Supplementary Table 2**. The mammalian signaling pathways analyzed in this work. Click here for file

Additional file 5 **Supplementary Table 3**. The result patterns of co-evolution for the small fungi dN/dS dataset. Click here for file

Additional file 6 **Supplementary Table 4**. Comparison between our approach and SAMBA for the small fungi dN/dS dataset. Click here for file

Additional file 7 **Supplementary Table 5**. The result patterns of co-evolution for the small fungi Copy Number dataset. Click here for file

Additional file 8 **Supplementary Table 6**. The result patterns of co-evolution for the small Eukaryote Copy Number dataset. Click here for file

Additional file 9 **Supplementary Table 7**. Relative levels of co-evolution of Fungal complexes along different parts of the evolutionary tree. Click here for file

Additional file 10 **Supplementary Table 8**. Relative levels of co-evolution of mammalian signaling pathways along different parts of the evolutionary tree. Click here for file
