# Supplementary material for: Discovering local patterns of co - evolution: computational aspects and biological examples
Source: BMC Bioinformatics. 2010 Jan 22;11:43. doi: 10.1186/1471-2105-11-43 (PMC3224649; doi:10.1186/1471-2105-11-43)
Supplement: Additional file 1 — Supplementary Note. 1 Hardness Issues. [file 1471-2105-11-43-S1.PDF]

Supplementary Material (Hardness Issues) for the work 'Discovering Local Patterns of Co - Evolution: Computational Aspects and Biological Examples'

Tamir Tuller, Yifat Felder, and Martin Kupiec

Tel Aviv University

This section deals with hardness issues which are related to the *Local Co-Evolutionary* problem. We show that some versions of the problem are NP-hard, but in practice it seems that the *Local Co-Evolutionary* problem has a shorter running time than the *bi-clustering* problem which is highly used in the context of gene expression analysis. Furthermore, we show that there are versions of the *Local Co-Evolutionary* problem that have a fixed-parameter tractable (FPT) or that are polynomial.

A *bi-cluster* is a subset of genes and a subset of conditions with the property that the selected genes are co-expressed (according to some measure of co-expression) in the selected conditions. Some versions of the *bi-clustering* problem are NP-hard (see for example [4, 1]). Let $D_b(S_g, S_c)$ denote a measure of co-expression of a set of genes, S_g, across a set of conditions, S_c. Formally, the *bi-clustering* problem is defined as follows:

Problem 1. Bi-clustering
Input: A set M of k vectors of length ℓ (each vector is related to one gene, and each component of the vectors is related to one condition), a measure of co-expression, $D_b(\cdot, \cdot)$, two natural numbers, p, q, and a positive real number c.
Question: Does the input include a set of p genes, S_g, and a set of q conditions, S_c, such that $D_b(S_g, S_c) < c$?

It is easy to see that the *bi-clustering* problem can be reduced to the *Local Co-Evolutionary* problem on trees with unbounded degree.

Lemma 1. *The* bi-clustering *problem can be reduced to the* Local Co-Evolutionary *problem on trees with unbounded degree.*

Proof. Given an input $< M, D_b, p, q, c >$ to the *Bi-clustering* problem generate the following input to the *Local Co-Evolution* problem: $n' = q$, $m' = p$, $d = c$, $D_b = D_c$, the tree T is a star where each of his edges is related to one of the components of the vectors in M, and $S = M$ (see Figure 3). It is easy to see that every subset of the conditions in M correspond to a subtree in T, proving the lemma.

However, in this work, we deal with binary trees. Unfortunately, the *Local Co-Evolutionary* problem is NP-hard also for binary trees. We prove it by reduction from the *Balanced Complete Bipartite Subgraph* problem that is known to be NP-Complete [2]:

Problem 2. Balanced Complete Bipartite Subgraph
Input: A bipartite graph, $G = (V_1, V_2, E)$, and a positive integer k.
Question: Are there two disjoint subset $V_1' \subseteq V_1, V_2' \subseteq V_2$ such that $|V_1'| = |V_2'| = k$ and $\forall_{v_1 \in V_1', v_2 \in V_2'} (v_1, v_2) \in E$.

Theorem 1. *The* Local Co-Evolution *on binary trees is NP-hard.*

Proof. We will prove Theorem 1 by reduction from the *Balanced Complete Bipartite Subgraph* problem.

Given an input $< G = (V_1, V_2, E), k >$ to the *Balanced Complete Bipartite Subgraph* problem we will generate the following input to the *Local Co-Evolution* problem, $< T, S, n', m', d, D_c >$:

- T is a tree with $|V_1| + |V_2|$ edges, an edge for each $v \in V_1 \cup V_2$.
- $S = [S_1, .., S_{|V_1|+|V_2|}]$ includes $|V_1| + |V_2|$ vectors of length $|V_1| + |V_2|$ (*i.e.* $\forall_i |S_i| = |V_1| + |V_2|$). A vector for each $v \in V_1 \cup V_2$, where $\forall_{i,j} S_{i,j} = \delta((i, j) \in E)$ (*i.e.* $|S|$ is the distance matrix of G).
- $n' = |V_1| + |V_2|$, $m' = k$, $d = |V_1| + |V_2| - k$.

– As a distance measure we use $D_c = D_{c3}$.

$\Longrightarrow$ Suppose G contains a balanced complete bipartite subgraph of size k, (V_1', V_2') (*i.e.* the answer to the *Balanced Complete Bipartite Subgraph* problem with $< G, k >$ is YES). By definition, each pair of vertices in the V_1' share k neighbors (the vertices in V_2'), thus, all the k vectors of evolutionary patterns that are related to the V_1' contain k common $'1'$s (the vertices that are in V_2'), *i.e.* $d = |V_1| + |V_2| - k$, and thus the answer to the *Local Co-Evolution* problem is YES.

$\Longleftarrow$ Suppose the answer to the *Local Co-Evolution* problem with $< T, S, n' = |V_1| + |V_2|, m' = k, d = |V_1| + |V_2| - k, D_{c3} >$ is YES. This means that there is a set of k edge orthologous labelings that share k positions with $'1'$ along the tree T, thus G includes two sets of k vertices each, such that all the vertices in one set (that is related to the k edge orthologous labelings) are connected to all the vertices in the second set (that is related to the k positions), *i.e.* balanced complete bipartite subgraph of size k.

Practically, due to the following lemma, it seems that the running time of the co-evolutionary problem should be shorter than the running time of the bi-clustering problem.

Lemma 2. *[3] The number of subtrees in a tree with n nodes is about* 1.48^n.

For example, suppose that we are dealing with n conditions in the case of the bi-clustering problem or tree of size n in the case of the *Local Co-Evolutionary* problem. The number of subgroups of n conditions is 2^n, while, by lemma 2, the number of connected subtrees of a tree with n nodes is only about 1.48^n. In practice, this can make a big difference, for example, if $n = 20$ there are less than $2,542$ connected subtrees while there are more than 10^6 subgroups of n conditions (a difference of three orders of magnitudes).

Similarly, considering a greedy procedure, there are at most n' ways to expand by one edge/node a subtree of size n', while in the general bi-clustering case, there are $n - n'$ such possibilities. This can make a big difference if $n' = o(1)$

Finally, there are versions of the co-evolutionary problem that are polynomial. For example, if we search exact co-evolution along paths (*i.e.* D_{c4}), or more generally along subtrees with k leaves [1] or k nodes. In this case, if k is constant, the number of such subtrees is polynomial.

Lemma 3. *The number of subtrees with k nodes in a tree with n nodes is less than* $(n-1) \cdot 2^k \cdot (k-1)!$.

Proof. We can generate (with repeats) all the subtrees with k nodes in the following way: Start with all the $n - 1$ subtrees of size two (all edges). In the i-th iteration, the number of trees of size i, T_i, is less than $T_{i-1} \cdot 2 \cdot i$ - all the possible additions of connected nodes to each of the trees from the previous step.

We say that an evolutionary pattern along a subtree T' is *supported* by a set of orthologous labelings, S', if there is $s \in S'$ with that evolutionary pattern along T'. Suppose that each component of an OL can have one of α possibilities. In this case, the number of possible evolutionary patterns that are supported by at least one of the OLs, in a tree with n nodes and when the input includes $|S|$ OLs is less than $min(|S| \cdot 1.48^n, \sum_{k=1}^{n}(n-1) \cdot (k-1)! \cdot 2^k \cdot \alpha^k)$. The left component is due to the fact that each of the 1.48^n subtrees can have maximum of S different evolutionary patterns, since each of the $|S|$ labeling induces only one pattern. The right components is an enumeration of all the possible patterns on all possible subtrees with k nodes ($0 < k \leq n$).

Thus, the problem of finding sets of OLs with the same pattern of evolution along subtrees of an evolutionary tree is a fixed-parameter tractable (FPT), which is exponential in the size of the tree, n (but not on the number of OLs). If we are interested in exact evolution along paths, *i.e.* subtrees with $k = 2$ leaves, this problem is polynomial since there are $O(n^2)$ paths, where each path can have at most $|S|$ evolutionary pattern, a total of $|S| \cdot n^2$ evolutionary patterns.

[1] Note that subtree with k leaves can includes $O(n)$ nodes. One such example is when the input tree is a path of length $n/2$ (two leaves, and $n-2$ internal nodes), and $n/2-2$ additional leaves that are connected to each of these $n-2$ internal nodes of the path, and when we seek co-evolution along paths (i.e. $k = 2$).

References

1. Amir Ben-Dor, Benny Chor, Richard Karp, and Zohar Yakhini. Discovering local structure in gene expression data: The order-preserving submatrix problem. *J. Comput. Biol.*, 10(3-4):373–384, 2003.
2. M.R. Garey and D.S. Johnsons. *Computers and Interactability: A Guide to the Theory of NP-Completeness*, page 196. Freeman. 1979.
3. B. Knudsen. Optimal multiple parsimony alignment with affine gap cost using a phylogenetic tree. *WABI03*, pages 433–446, 2003.
4. Amos Tanay, Roded Sharan, and Ron Shamir. Discovering statistically significant biclusters in gene expression data. *Bioinformatics*, 18:S136–44, 2002.
